# Streptinone, a New Indanone Derivative from a Marine-Derived *Streptomyces massiliensis,* Inhibits Particulate Matter-Induced Inflammation

**DOI:** 10.3390/md21120640

**Published:** 2023-12-14

**Authors:** Hwa-Sun Lee, Dineth Pramuditha Nagahawatta, You-Jin Jeon, Min Ah Lee, Chang-Su Heo, Sun Joo Park, Hee Jae Shin

**Affiliations:** 1 Marine Natural Products Laboratory, Korea Institute of Ocean Science and Technology, 385 Haeyang-ro, Yeongdo-gu, Busan 49111, Republic of Korea; hwasunlee@kiost.ac.kr (H.-S.L.); minah@kiost.ac.kr (M.A.L.); science30@kiost.ac.kr (C.-S.H.); 2 Department of Chemistry, Pukyong National University, 45 Yongso-ro, Nam-gu, Busan 48513, Republic of Korea; parksj@pknu.ac.kr; 3 Department of Marine Life Sciences, Jeju National University, 102 Jeju Daehak-ro, Jeju 63243, Republic of Korea; dineth1673@gmail.com (D.P.N.); youjin2014@gmail.com (Y.-J.J.); 4 Department of Marine Biotechnology, University of Science and Technology (UST), 217 Gajung-ro, Yuseong-gu, Daejeon 34113, Republic of Korea

**Keywords:** *Streptomyces massilinesis*, indanone, particulate matter, anti-inflammation

## Abstract

Inflammatory diseases caused by air pollution, especially from particulate matter (PM) exposure, have increased daily. Accordingly, attention to treatment or prevention for these inflammatory diseases has grown. Natural products have been recognized as promising sources of cures and prevention for not only inflammatory but also diverse illnesses. As part of our ongoing study to discover bioactive compounds from marine microorganisms, we isolated streptinone, a new indanone derivative (**1**), along with three known diketopiperazines (**2**–**4**) and piericidin A (**5**), from a marine sediment-derived *Streptomyces massiliensis* by chromatographic methods. The structure of **1** was elucidated based on the spectroscopic data analysis. The relative and absolute configurations of **1** were determined by ^1^H-^1^H coupling constants, 1D NOESY, and ECD calculation. The anti-inflammatory activities of **1** were evaluated through enzyme-linked immunosorbent assay (ELISA), Western blot, and qPCR. Compound **1** suppressed the production of nitric oxide (NO), prostaglandin E_2_ (PGE_2_), and pro-inflammatory cytokines such as TNF-*α*, IL-6, and IL-1*β*, by inhibiting the Toll-like receptor (TLR)-mediated nuclear factor kappa B (NF-*κ*B) signaling pathway. Therefore, compound **1** could potentially be used as an agent in the prevention and treatment of diverse inflammatory disorders caused by particulate matter.

## 1. Introduction

**Figure 1:**
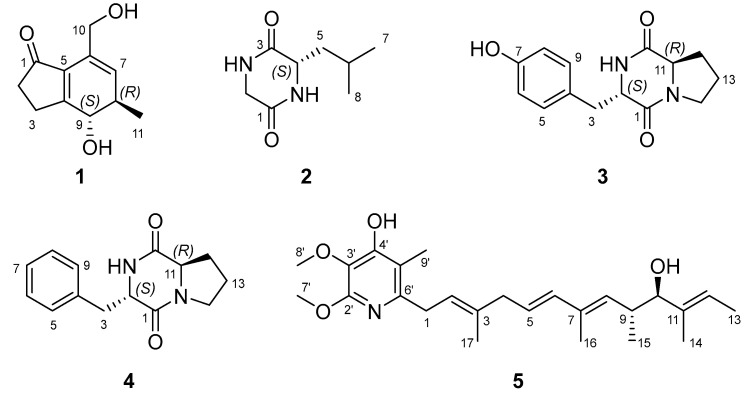
Structures of **1**–**5** isolated from *Streptomyces massiliensis* 213DD-128.

Particulate matter (PM) is considered representative of air pollution and is generated from natural (volcanos, forest fires, and desert dust), social (traffic, industry, and agriculture fields), and personal (cooking, heating, and smoking) sources [1]. Recently, exposure to PM consisting of toxic substances has rapidly increased and it causes diverse diseases related to the cardiovascular, respiratory, reproductive, and brain systems [2]. PM can induce oxidative stress and stimulate pro-inflammatory cytokines such as TNF-*α*, IL-6, and IL-1*β* [3]. Therefore, the need for drugs for diseases related to PM has increased, and natural products (NPs) are still recognized as one of the attractive sources for the prevention and treatment of inflammatory diseases. Overall, 31.6% of approved small-molecule medicines have been reported as derived from NPs or their derivatives [4]. As part of our ongoing study to discover bioactive compounds from marine microbes, we found a new indanone derivative (**1**, Figure 1) exhibiting an inhibitory effect on PM-induced inflammation from *Streptomyces massiliensis* isolated from a sediment sample collected from the East Sea, Republic of Korea. In 2013, *Streptomyces massiliensis* was first reported as a new species from the human gut [5]. Since then, natural product studies on *S. massiliensis* have not been reported. Only one paper described the anti-bacterial activities of *S. massiliensis* against Gram-positive and Gram-negative bacteria [6]. Thus, this is the first report on the bioactive compounds from *S. massiliensis*.

Indanone exists as two isomeric benzocyclopentanones and its derivatives were isolated from the cyanobacterium *Lyngbya majuscule* [7] and plants (*Pteridium aquilinum* [8], *Sesamum alatum* [9], *Vatica pauciflora* [10], and *Pterius multifida* [11]). Some compounds bearing indanone as a substructure have been also reported from the marine sponge *Terpios hoshinota* [12], the bacterium *Streptomyces rosa* [13]*,* the fungus *Nodulisporium* sp. [14], and some plants [15,16]. Indanone derivatives are reported to have anti-proliferative [9], cytotoxic [17], anti-inflammatory [18,19], anti-acetylcholinesterase [19], anti-oxidant [19], neuroprotective [20], and anti-osteoporotic [21] activities. Herein, we describe the isolation, purification, and structure elucidation of the new indanone derivative (**1**) and its inhibitory activity against PM-induced inflammation in RAW 264.7 cells.

## 2. Results and Discussion

**Figure 2:**
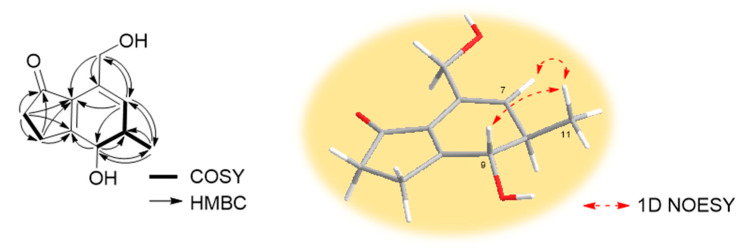
Key COSY, HMBC, and 1D NOESY correlations of **1**.

**Figure 3:**
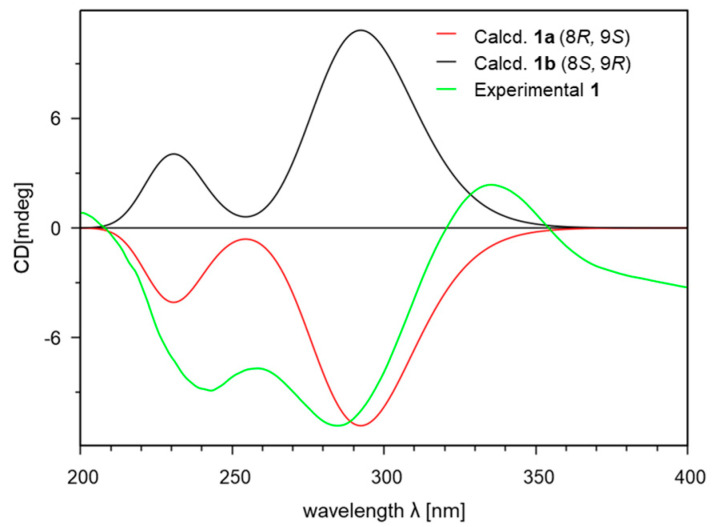
Experimental and calculated ECD spectra of **1**.

Compound **1** was isolated as a yellowish amorphous powder. The molecular formula C_11_H_14_O_3_ was deduced from the [M + Na]^+^ peak at *m*/*z* 217.0841 (C_11_H_14_O_3_Na, calculated for 217.0841, Figure S8) in the HR-ESIMS spectrum, which required five degrees of unsaturation. The IR spectrum exhibited characteristic absorption bands at 3322 (O-H), 1674 (C=O), and 1600 (C=C) cm^−1^. The ^1^H (Table 1 and Figure S1) and HSQC (Figure S3) NMR spectra of **1** showed one olefin proton at *δ*_H_ 5.70 (br s, H-7); one oxygenated methine proton at *δ*_H_ 4.26 (d, *J* = 12.2 Hz, H-9); one oxygenated methylene proton at *δ*_H_ 4.33 (d, *J* = 13.6 Hz, Ha-10) and 4.29 (d, *J* = 13.6 Hz, Hb-10), one methine proton at *δ*_H_ 2.54 (m, overlapped, H-8); two methylene protons at *δ*_H_ 2.93 (m, H-3a), 2.66 (m, H-3b), and 2.52 (t, overlapped, H_2_-2); and one methyl proton at *δ*_H_ 1.20 (d, *J* = 7.2 Hz, H_3_-11). The ^13^C (Table 1 and Figure S2) and HSQC NMR spectra of **1** revealed the presence of one ketone carbon at *δ*_C_ 209.7 (C-1); three non-protonated sp^2^ carbons at *δ*_C_ 180.0 (C-4), 136.2 (C-5), and 132.5 (C-6); one protonated sp^2^ carbon at *δ*_C_ 131.2 (C-7); one oxygenated methine sp^3^ carbon at *δ*_C_ 75.0 (C-9); one oxygenated methylene sp^3^ carbon at *δ*_C_ 62.9 (C-10); one methine carbon at *δ*_C_ 39.3 (C-8); two methylene carbons at *δ*_C_ 36.9 (C-2) and 27.6 (C-3); and one methyl carbon at *δ*_C_ 18.4 (C-11). The analysis of the COSY spectrum (Figure S4) suggested two spin systems (Figure 2): from H_2_-2 (*δ*_H_ 2.52) to H-3a (*δ*_H_ 2.93) and H-3b (*δ*_H_ 2.66), from H-7 (*δ*_H_ 5.70) to H-9 (*δ*_H_ 4.26), and from H-8 (*δ*_H_ 2.54) to H-11 (*δ*_H_ 1.20). The position of ketone was determined by HMBC correlations (Figure 2 and Figure S5) from H_2_-2 (*δ*_H_ 2.52)/H-3a (*δ*_H_ 2.93)/H-3b (*δ*_H_ 2.66) to C-1 (*δ*_C_ 209.7). The position of oxygenated methylene was identified by HMBC correlations (Figure 2 and Figure S5) from H-10a (*δ*_H_ 4.33)/H-10b (*δ*_H_ 4.29) to C-5 (*δ*_C_ 136.2)/C-6 (*δ*_C_ 132.5)/C-7 (*δ*_C_ 131.2). The linkage of two partial structures was confirmed by HMBC correlations (Figure 2 and Figure S5) from H_2_-2 (*δ*_H_ 2.52)/H-3a (*δ*_H_ 2.93)/H-3b (*δ*_H_ 2.66)/H-7 (*δ*_H_ 5.70) to C-5 (*δ*_C_ 136.2) and from H_2_-2 (*δ*_H_ 2.52)/H-3a (*δ*_H_ 2.93)/H-3b (*δ*_H_ 2.66)/H-9 (*δ*_H_ 4.26) to C-4 (*δ*_C_ 180.0). These connections suggested that the remaining two degrees of unsaturation are due to the 1-indanone ring system having five- and six-membered rings. An *anti*-relationship between the methyl group and the 9-hydroxy group was established on the basis of the large ^3^*J*_H8/H9_ value (12.2 Hz). The 1D NOESY correlations (Figures S6 and S7) between H-7 (*δ*_H_ 5.70) and H_3_-11 (*δ*_H_ 1.20), between H_3_-11 (*δ*_H_ 1.20) and H-9 (*δ*_H_ 4.26) also supported this relative configuration (Figure 2). To determine the absolute configuration of **1**, the theoretical electronic circular dichroism (ECD) spectra of two possible diastereomers (8R*, 9S* and 8S*, 9R*) were calculated by Gaussian 16 and compared with the experimental ECD spectrum (Figure 3). The experimental ECD spectrum of **1** was similar to that of an 8*R*, 9*S*-isomer (**1a**), and the absolute configuration of **1** was determined to be 8*R*, 9*S*. Thus, the gross structure of **1** was determined to be a new derivative of indanone as shown in Figure 1, and **1** was named streptinone.

The structures of the known compounds **2**–**5** (Figure 1) were identified as cyclo(Gly-L-Leu) (**2**, Figure S9) [22], cyclo(L-Tyr-D-Pro) (**3**, Figure S10) [23], cylco(L-Phe-D-Pro) (**4**, Figure S11) [24], and piericidin A (**5**, Figure S12) [25] by the comparison of their spectroscopic data with those reported in the literature.

**Figure 4:**
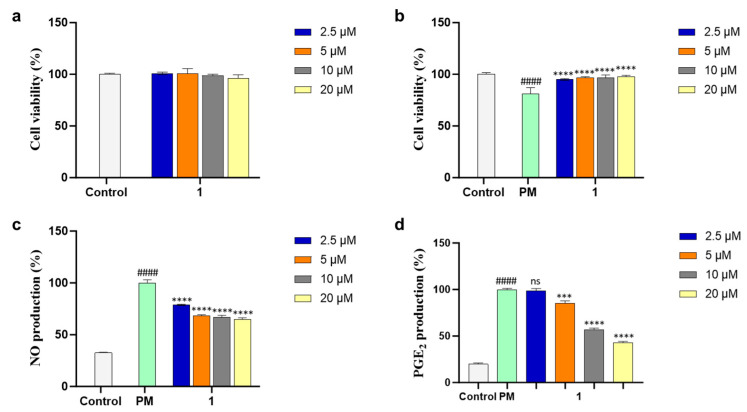
(**a**) Cytotoxicity, (**b**) cytoprotective, (**c**) nitric oxide (NO), and (**d**) prostaglandin E_2_ (PGE_2_) inhibitory effects of **1** in particulate matter (PM)-induced RAW 264.7 macrophages. Experiments were performed in triplicate and the results are represented as means ± SD. Values are significantly different from the particulate matter (PM)-treated group (*** *p* < 0.005 and **** *p* < 0.0001) and from the control group (#### *p* < 0.0001). ns is short for not significant.

Compound **1** was evaluated for an inhibitory effect on PM-induced inflammation. The cell viability of **1** was measured by MTT assay in various concentrations (2.5, 5, 10, and 20 µM) to select the safe dose range for further experiments and **1** did not show any cytotoxic effect on RAW 264.7 cells (Figure 4a). The cell-protective effect of **1** against PM-stimulated RAW 264.7 cells was evaluated in the same manner and the cell viability of PM-treated RAW 264.7 cells was slightly decreased. On the other hand, the cells treated with **1** exhibited similar cell viability compared with control (Figure 4b). These results revealed the protective activity of **1** against PM-induced inflammation.

PM induces the production of the main inflammatory mediators NO and PGE_2_ and leads to inflammatory diseases. Thus, the NO and PGE_2_ inhibitory activities of **1** were assessed in the PM-activated RAW 264.7 cells, and **1** reduced levels of NO and PGE_2_ production in a dose-dependent manner (Figure 4c,d). These results suggested the inhibitory effect of **1** on PM-induced inflammation and led to further mechanism studies.

**Figure 5:**
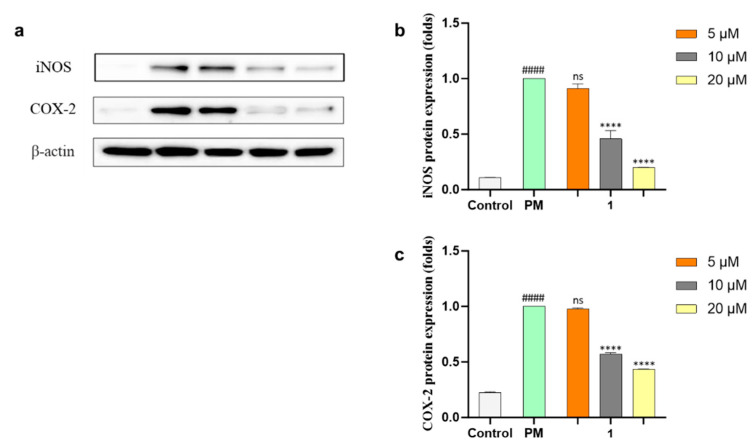
(**a**) Western blot results of inducible nitric oxide (iNOS) and cyclooxygenase-2 (COX-2) protein expressions. *β*-actin was used as an internal reference. (**b**,**c**) Quantification of iNOS and COX-2. Experiments were performed in triplicate and the results are represented as means ± SD. Values are significantly different from the particulate matter (PM)-treated group (**** *p* < 0.0001) and from the control group (#### *p* < 0.0001). ns is short for not significant.

**Figure 6:**
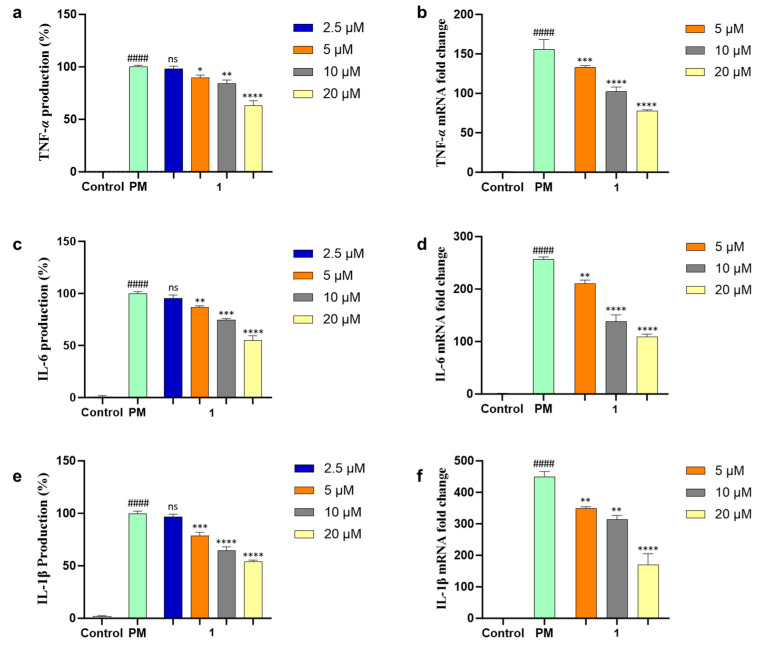
Inhibition effect of **1** in PM-induced RAW 264.7 cells on pro-inflammatory cytokines (**a**) TNF-*α* production, (**b**) mRNA expression of TNF-*α*, (**c**) IL-6 production, (**d**) mRNA expression of IL-6, (**e**) IL-1*β* production, and (**f**) mRNA expression of IL-1*β*. Experiments were performed in triplicate and the results are represented as means ± SD. Values are significantly different from the particulate matter (PM)-treated group (* *p* < 0.05, ** *p* < 0.01,*** *p* < 0.005 and **** *p* < 0.0001) and from the control group (#### *p* < 0.0001). ns is short for not significant.

**Figure 7:**
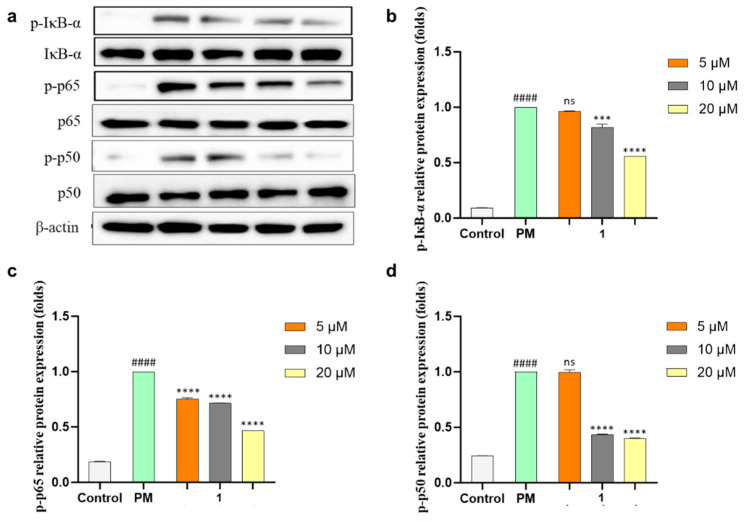
Inhibitory effects of **1** on Toll-like receptor (TLR)-mediated nuclear factor *κ*B (NF-*κ*B)-associated protein expression in PM-induced RAW 264.7 cells. (**a**) Expression analysis of inhibitor *κ*B alpha (I*κ*B-*α*), p65 and p50, and phosphorylation in cytosol and nucleus evaluated using Western blotting after the treatment of **1** in PM-stimulated macrophages. Relative protein expression of (**b**) p-I*κ*B-*α*, (**c**) p-p65, and (**d**) p-p50. Experiments were performed in triplicate and the results are represented as means ± SD. Values are significantly different from the particulate matter (PM)-treated group (*** *p* < 0.005 and **** *p* < 0.0001) and from the control group (#### *p* < 0.0001). ns is short for not significant.

NO and PGE_2_ are related to two enzymes, inducible nitric oxide (iNOS) and cyclooxygenase-2 (COX-2), which mediate inflammatory processes. To confirm the anti-inflammatory effects of **1**, the protein expressions of iNOS and COX-2 under the PM-stimulated cells were evaluated with Western blot analysis and both protein expressions were downregulated dose-dependently (Figure 5) by **1**. The production of pro-inflammatory cytokines (TNF-*α*, IL-6, and IL-1*β*) in PM-induced RAW 264.7 cells was evaluated using ELISA kits. Compound **1** significantly suppressed the level of pro-inflammatory cytokine secretion at concentrations of more than 5 µM (Figure 6a,c,e). The mRNA expressions of TNF-*α*, IL-6, and IL-1*β* in PM-stimulated RAW 264.7 cells were evaluated with qPCR. As a result, compound **1** successfully inhibited the gene expression of these cytokines (Figure 6b,d,f). Furthermore, **1** was tested using Western blot analysis on major inflammatory pathway signals related to PM-induced Toll-like receptor (TLR)-mediated NF-*κ*B. The phosphorylation levels of I*κ*B-*α*, p65, and p50 in the cytosol increased with PM activation as compared with control cells. Treatment of **1** decreased the phosphorylation and nuclear translocation of I*κ*B-*α*, p65, and p50 (Figure 7). These results suggested that **1** suppresses the PM-stimulated inflammatory responses in the RAW 264.7 cells by inhibiting the phosphorylation of proteins, such as I*κ*B-*α*, p65, and p50, related to the TLR-mediated NF-*κ*B pathway.

## 3. Materials and Methods

### 3.1. General Experimental Procedures and Reagents

Optical rotations were obtained on a Rudolph analytical Autopol Ⅲ S2 polarimeter (Rudolph Research Analytical, Hackettstown, NJ, USA) with a sodium D line at 589 nm and 10 mm path length. UV spectra were measured with a Shimadzu UV-1650PC spectrophotometer (Shimadzu Corporation, Kyoto, Japan). IR spectra were recorded by a Bruker ALPHA Ⅱ spectrophotometer (Bruker OPTIK GmbH & Co. KG, Ettlingen, Germany). ECD spectra were recorded with a JASCO J-1500 circular dichroism spectrophotometer (JASCO Corporation, Tokyo, Japan) at the Center for Research Facilities, Changwon National University in Changwon, Republic of Korea. NMR spectra were measured with a Bruker AVANCE Ⅲ 600 spectrometer (Bruker Biospin GmbH, Rheinstetten, Germany) with a 3 mm probe operating at 600 MHz (^1^H) and 150 MHz (^13^C). Chemical shifts were referenced to the residual solvent peaks (*δ*_H_ 3.31 and *δ*_C_ 49.15 ppm for CD_3_OD, *δ*_H_ 7.24 and *δ*_C_ 77.23 ppm for CDCl_3_). HR-ESIMS data were obtained with a Waters SYNPT G2 Q-TOF mass spectrometer (Waters Corporation, Milford, MA, USA) at the Korea Basic Science Institute (KBSI) in Cheongju, Republic of Korea. HPLC was performed using a PrimeLine binary pump (Analytical Scientific Instruments, Inc., El Sobrante, CA, USA) with a Shodex RI-101 refractive index detector (Shoko Scientific Co., Ltd., Yokohama, Japan) and S3210 variable UV detector (Schambeck SFD GmbH, Bad Honnef, Germany). Columns used for HPLC were YMC-Triart C_18_ (250 mm × 10 mm, i.d, 5 μm and 250 mm × 4.6 mm, i.d, 5 μm) and YMC-Pack SIL (250 mm × 10 mm, i.d, 5 μm). Reversed-phase silica gel (YMC-Gel ODS-A, 12 nm, S-75 μm) was used for open-column chromatography. Organic solvents for extraction and purification of compounds were purchased as extra-pure (EP) or HPLC-grade from Samchun (Pyeongtaek, Republic of Korea). Pure and ultrapure water was used from the Milipore Mili-Q Direct 8 system (Milipore S.A.S, Molsheim, France). 

The RAW 264.7 cell line was acquired from the American Type Culture Collection (ATCC, Rockville, MD, USA). Dulbecco’s modified Eagle’s medium (DMEM), fetal bovine serum (FBS), and penicillin–streptomycin (PS) were obtained from Gibco-BRL (Burlington, ON, Canada). The urban particulate matter (PM) was sourced from the National Institute for Environmental Studies (Ibaraki, Japan). Enzyme-linked immunosorbent assay (ELISA) kits for tumor necrosis factor-*α* (TNF-*α*), prostaglandin E_2_ (PGE_2_), interleukin-6 (IL-6), and interleukin-1*β* (IL-1*β*) were procured from R&D Systems Inc. (Minneapolis, MN, USA). 3-(4,5-dimethylthiazol-2-yl)-2,5-diphenyltetrazolium bromide (MTT), 2-propanol, ethanol, bicinchoninic acid (BCA) protein assay kits, and chloroform were purchased from Sigma-Aldrich (St. Louis, MO, USA). The primary and secondary antibodies were obtained from Santa Cruz Biotechnology (Dallas, TX, USA). Enhanced chemiluminescence reagents were used from Amersham Life Science Inc. (Arlington Heights, IL, USA). All other chemicals and solvents for assay utilized in this study were of analytical grade.

### 3.2. Strain and Fermentation

The strain 213DD-128 was isolated from a sediment sample collected using a grab sampler mounted on the R/V ONNURI from the East Sea, Republic of Korea (date: 1 April 2021, latitude: 37°13′24.71″ N, longitude: 131°52′03.54″ E, depth: 127 m). A sediment sample heated at 60 °C for 30 min was spread onto the 10% Bennett (BN)’s agar plates (0.1% glucose, 0.02% tryptone, 0.01% yeast extract, 0.01% beef extract, 0.05% glycerol, 3.2% artificial sea salt, 1.8% agar, and pH 7.02 before sterilization). The plates were incubated for three weeks at 28 °C in a B.O.D incubator. The color of the colonies changed from beige to dark brown and the strain formed grey spores. As time went by, the color of the BN agar medium turned brown. The strain was identified as *Streptomyces massiliensis* on the basis of 16S rRNA sequence analysis by Macrogen Inc. (Seoul, Republic of Korea). The sequence of 213DD-128 was submitted to GenBank under accession number OR807412. 

The seed and production cultures of the strain 213DD-128 were conducted using the BN broth medium (1% glucose, 0.2% tryptone, 0.1% yeast extract, 0.1% beef extract, 0.5% glycerol, 3.2% artificial sea salt, and pH 7.02 before sterilization). The seed cultures were performed in two 100 mL flasks each containing 50 mL of BN broth medium at 28 °C for 8 days on a rotary shaker at 140 rpm. An aliquot (0.2%, *v*/*v*) from the seed culture broth was inoculated into nine 2 L flasks each containing 700 mL of BN broth in an aseptic, and cultivated at 28 °C for 26 days on a rotary shaker at 120 rpm. 

### 3.3. Extraction and Isolation of Compounds

The cultivated broth was extracted with twice equal volumes of ethyl acetate (EtOAc, 7 L × 2). The EtOAc layer was evaporated to obtain a crude extract (284.8 mg). The crude extract was applied to reversed-phase vacuum column chromatography (YMC Gel ODS-A, 12 nm, S 75 μm) with a stepwise gradient solvent system of 20, 40, 60, 80, and 100% MeOH in H_2_O. The 40% MeOH fraction (47.2 mg) was purified by reversed-phase HPLC (YMC, Triart C_18_, 250 × 10 mm, 5 μm, 15% MeCN in H_2_O, flow rate: 2.0 mL/min, detector: RI) to give **1** (6.8 mg, *t*_R_ 18 min) and **4** (0.4 mg, *t*_R_ 39 min). The 20% MeOH fraction (63.6 mg) was applied to reversed-phase HPLC (YMC, Triart C_18_, 250 × 10 mm, 5 μm, 12% MeOH in H_2_O, flow rate: 2.0 mL/min, detector: RI) to obtain **2** (1.6 mg, *t*_R_ 50 min), and **3** (2.5 mg, *t*_R_ 67 min). The 100% MeOH fraction (74 mg) was purified by normal-phase HPLC (YMC-pack SIL, 250 × 10 mm, 5 μm, 25% EtOAc in Hex, flow rate: 2.0 mL/min, detector: RI) to get **5** (25.6 mg, *t*_R_ 11 min).

Streptinone (**1**): yellowish amorphous; [*α*${]}_{D}^{25}$ −20 (*c* 0.1, MeOH); UV (MeOH) *λ*_max_ (log *ε*) 230 (1.06), 268 (0.75) nm; ECD (1.3 µM, MeOH) *λ*_max_ (*Δε*) 385 (+0.20), 285 (−0.90), 243 (−0.74), 200 (+0.07) nm; IR (MeOH) *γ*_max_ 3322, 2927, 1674, 1600 cm^−1^; ^1^H and ^13^C NMR data, Table 1; HR-ESIMS *m/z* 217.0841 [M + Na]^+^ (calculated for C_11_H_14_O_3_Na, 217.0841).

Cyclo(Gly-L-Leu) (**2**): ^1^H NMR (CD_3_OD) *δ*_H_ 4.00 (1H, dd, *J* = 17.8, 0.6 Hz, H-2), 3.89 (1H, dd, *J* = 8.1, 5.6 Hz, H-4), 3.83 (1H, d, *J* = 17.8 Hz, H-2′), 1.83 (1H, m, H-6), 1.70-1.66 (2H, m, H-5), 0.98 (3H, d, *J* = 6.6 Hz, H-7 or H-8), and 0.96 (3H, d, *J* = 6.6 Hz, H-7 or H-8). 

Cyclo(L-Tyr-D-Pro) (**3**): ^1^H NMR (CD_3_OD) *δ*_H_ 6.98 (2H, d, *J* = 8.5 Hz, H-5, H-9), 6.72 (2H, d, *J* = 8.5 Hz, H-6, H-8), 4.14 (1H, t, *J* = 4.3 Hz, H-2), 3.56-3.51 (1H, m, H-14), 3.32-3.29 (1H, overlapped, H-14′), 3.11 (1H, dd, *J* = 13.9, 4.3 Hz, H-3), 2.87 (1H, dd, *J* = 13.9, 4.6 Hz, H-3′), 2.61 (1H, dd, *J* = 10.5, 6.5 Hz, H-11), 2.06 (1H, m, H-12), 1.91 (1H, m, H-13), and 1.70-1.60 (2H, overlapped, H-12′, H-13′).

Cyclo(L-Phe-D-Pro) (**4**): ^1^H NMR (CD_3_OD) *δ*_H_ 7.31-7.30 (3H, overlapped, H-6, H-7, and H-8), 7.19-7.18 (2H, overlapped, H-5, H-9), 4.20 (1H, t, *J* = 4.7 Hz, H-2), 3.54 (1H, m, H-14), 3.34 (1H, overlapped with H_2_O, H-14′), 3.19 (1H, dd, *J* = 13.7, 4.8 Hz, H-3), 2.99 (1H, dd, *J* = 13.7, 4.7 Hz, H-3′), 2.62 (1H, dd, *J* = 10.6, 6.4 Hz, H-11), 2.03 (1H, m, H-12), 1.90 (1H, m, H-13), and 1.69-1.60 (2H, overlapped, H-12′, H-13′).

Piericidin A (**5**): ^1^H NMR (CDCl_3_) *δ*_H_ 6.06 (1H, d, *J* = 15.5 Hz, H-6), 5.58 (1H, dt, *J* = 15.5, 7.0 Hz, H-5), 5.45 (1H, q, *J* = 12.5, 6.2 Hz, H-12), 5.39 (1H, dt, *J* = 7.0, 1.0 Hz, H-2), 5.19 (1H, d, *J* = 9.7 Hz, H-8), 3.92 (3H, s, H-7′), 3.81 (3H, s, H-8′), 3.60 (1H, d, *J* = 9.1 Hz, H-10), 3.35 (2H, d, *J* = 6.9 Hz, H-1), 2.76 (2H, d, *J* = 6.9 Hz, H-4), 2.69-2.63 (1H, m, H-9), 2.07 (3H, s, H-9′), 1.78 (3H, d, *J* = 1.0 Hz, H-16), 1.72 (3H, s, H-17), 1.61 (3H, s, H-14), 1.60 (3H, d, *J* = 7.3 Hz, H-13), and 0.78 (3H, d, *J* = 6.7 Hz, H-14).

### 3.4. Computational Analysis

The initial geometry optimization and conformational searches of **1** were carried out using the Conflex 8 (Rev. B, Conflex Corp., Tokyo, Japan). The optimization and calculation for electronic circular dichroism (ECD) were performed using the Gaussian 16 (rev. B.01, Gaussian Corp., Wallingford, CT, USA). Conformational searches were performed using molecular mechanics force-field (MMFF94s) calculations with a search limit of 5 kcal/mol. The low-energy conformers of **1**, which accounted for more than 1%, were optimized using the density functional theory (DFT) method at the CAM-B3LYP/6-31+G(d,p) level in MeOH with the polarizable continuum model (PCM) using the integral equation formalism variant (IEFPCM) model for ECD calculation. The theoretical calculations of ECD spectra were performed using TD-SCF and the DFT method at the CAM-B3LYP/6-31+G(d,p) level in MeOH with the IEFPCM model. The calculated ECD spectra were combined and simulated by SpecDis (v. 1.71) [26] using *σ* = 0.40 eV. Calculated curves were shifted to 12 nm to adjust based on the experimental UV spectrum.

### 3.5. Cell Culture and Cell Viability Evaluation

The RAW 264.7 cells were cultured in 10% FBS and 1% PS-supplemented DMEM cell culture media. The cultured cells were maintained in a controlled environment consisting of 5% CO_2_ at 37 °C. The cell line was periodically sub-cultured, and cells in exponential growth were utilized for subsequent experiments. The cell viability was measured by MTT assay using the previously optimized method [27]. 

### 3.6. Cytoprotectivity and NO-Inhibitory Activity Evaluation

Cells were seeded in 48 wells in a cell culture plate, and cells were treated with samples after 24 h of incubation. Then, cells were stimulated by 125 μg/mL of PM after a 2 h incubation period. Finally, the cell viability was measured by MTT assay [27]. PM-induced NO production in RAW 264.7 cells was measured by Griess assay [28]. 

### 3.7. Evaluation of PGE_2_ and Levels of Pro-Inflammatory Cytokines in PM-Stimulated RAW 264.7 Cells

Cells were seeded in 48 wells in a cell culture plate and the cell culture supernatant was collected for subsequent ELISA assay. PGE_2_, TNF-*α*, IL-6, and IL-1*β* ELISA kits were used for analyzing each cytokine expression according to the instructions from the manufacturer.

### 3.8. Gene Expression Analysis

Total RNA extraction was performed using PM-stimulated cells treated with **1**. TRIzol reagent (Life Technologies, Carlsbad, CA, USA) was utilized for total RNA extraction according to the formerly established method [28]. 

The complementary cDNA was generated by the cDNA Reverse Transcription Kit (Takara Bio Inc., Shiga, Japan). The Thermal Cycler Dice Real-Time System (Takara Bio Inc., Kusatsu, Shiga, Japan) was utilized with the following protocol: initial enzyme activation at 95 °C for 5 s, followed by 40 cycles involving denaturation at 95 °C for 5 s and annealing at 58 °C for 10 s. The reaction was conducted in a 10 µL volume, comprising 3 µL of cDNA, 5 µL of the 2× TaKaRa ExTaq SYBR premix, 0.4 µL of each primer, and 1.2 µL of RNase/DNase-free water. GAPDH was employed as an internal reference standard gene during the amplification process [27]. The primers utilized for qPCR are summarized in Table 2.

### 3.9. Protein Expression Evaluation

Protein expression of iNOS, COX-2, and NF-*κ*B signaling pathways were evaluated using Western blot. Cells were harvested by PBS and lysed with RIPA buffer. Subsequent homogenates were centrifuged (12,000 rpm, 20 min), and protein content in the supernatant was measured. An equal amount (30 μg) of protein was loaded into each well and subjected to electrophoresis on 10% sodium dodecyl sulfate-polyacrylamide gel. The gel was transferred onto a nitrocellulose membrane (Bio-Rad, Hercules, CA, USA). The Western blot analysis was performed according to a previously described method [29].

### 3.10. Statistical Analysis

To derive the ultimate dataset, the experiments were conducted in triplicate, and the data were presented using means and standard deviations. Statistical significance was evaluated through one-way analysis of variance (ANOVA) combined with Tukey’s test. For comparisons involving two conditions, two-tailed Student’s t-tests were employed. The significance threshold was established at *p* < 0.05. All statistical analyses were executed using GraphPad Prism 10 software (San Diego, CA, USA).

## 4. Conclusions

Streptinone, a new indanone derivative (**1**), three known diketopiperazines, including cyclo(Gly-L-Leu) (**2**), cyclo(L-Tyr-D-Pro) (**3**), and cyclo(L-Phe-D-Pro) (**4**), and the previously reported piericidin A (**5**) were isolated from the EtOAc extract of *Streptomyces massiliensis* 213DD-128 culture broth by chromatographic methods. The structure of **1** was elucidated by the interpretation of NMR and HR-ESIMS spectral data. The relative and absolute configurations of **1** were determined by ^1^H-^1^H coupling constants, 1D NOESY, and ECD calculation. Streptinone (**1**) was evaluated for its inhibitory effect on PM-induced inflammation in RAW 264.7 cells through ELISA, Western blot, and qPCR. The levels of inflammatory products were decreased by **1** by regulating the expression of iNOS, COX-2, TNF-*α*, IL-6, and IL-1*β*. Additionally, **1** downregulated phosphorylation and nuclear translocation of I*κ*B-*α*, p65, and p50. According to these results, the inhibitory effect of **1** on TLR-mediated NF-*κ*B pathway signals was confirmed, revealing that **1** has the potential to be used as an agent for prevention and treatment in PM-stimulated inflammation-related diseases. 

## Figures and Tables

**Table 1:** ^1^H and ^13^C NMR data for **1** in CD_3_OD.

No	*δ*_C_, Type	*δ*_H_, Mult. (*J* in Hz)
1	209.7, C	
2	36.9, CH_2_	2.52 t (overlapped)
3	27.6, CH_2_	2.93 m 2.66 m
4	180.0, C	
5	136.2, C	
6	132.5, C	
7	131.2, CH	5.70 br s
8	39.3, CH	2.54 m (overlapped)
9	75.0, CH	4.26 d (12.2)
10	62.9, CH_2_	4.33 d (13.6) 4.29 d (13.6)
11	18.4, CH_3_	1.20 d (7.2)

**Table 2:** Primer sequences.

No	Primer Name	Sequence
1	GAPDH forward	5′-AAGGGTCATCATCTCTGCCC-3′
2	GAPDH reverse	5′-GTGATGGCATGGACTGTGGT-3′
3	TNF-*α* forward	5′-TTGACCTCAGCGCTGAGTTG-3′
4	TNF-*α* reverse	5′-CCTGTAGCCCACGTCGTAGC-3′
5	IL-6 forward	5′-GTACTCCAGAAGACCAGAGG-3′
6	IL-6 reverse	5′-TGCTGGTGACAACCACGGCC-3′
7	IL-1*β* forward	5′-CAGGATGAGGACATGAGCACC-3′
8	IL-1*β* reverse	5′-CTCTGCAGACTCAAACTCCAC-3′

## Data Availability

The data presented in this study are available in Supplementary Material.

## Supplementary Materials

The following supporting information can be downloaded at: https://www.mdpi.com/article/10.3390/md21120640/s1, Figures S1–S8: 1D, 2D NMR, and HR-ESIMS data of **1**; Figures S9–S12: ^1^H NMR spectra of cyclo(Gly-L-Leu) (**2**), cyclo(L-Tyr-D-Pro) (**3**), cyclo(L-Phe-D-Pro) (**4**), and piericidin A (**5**).

## References

[1] Bhatnagar A. (2022). Cardiovascular Effects of Particulate Air Pollution. Annu. Rev. Med..

[2] Thangavel P., Park D., Lee Y.C. (2022). Recent Insights into Particulate Matter (PM)-Mediated Toxicity in Humans: An Overview. Int. J. Environ. Res. Public Health.

[3] Hantrakool S., Kumfu S., Chattipakorn S.C., Chattipakorn N. (2022). Effects of Particulate Matter on Inflammation and Thrombosis: Past Evidence for Future Prevention. Int. J. Environ. Res. Public Health.

[4] Newman D.J., Cragg G.M. (2020). Natural Products as Sources of New Drugs over the Nearly Four Decades from 01/1981 to 09/2019. J. Nat. Prod..

[5] Pfleiderer A., Lagier J.C., Armougom F., Robert C., Vialettes B., Raoult D. (2013). Culturomics identified 11 new bacterial species from a single anorexia nervosa stool sample. Eur. J. Clin. Microbiol. Infect. Dis..

[6] Fritz S., Rajaonison A., Chabrol O., Raoult D., Rolain J.M., Merhej V. (2018). Full-length title: NRPPUR database search and in vitro analysis identify an NRPS-PKS biosynthetic gene cluster with a potential antibiotic effect. BMC Bioinform..

[7] Nagle D.G., Zhou Y.D., Park P.U., Paul V.J., Rajbhandari I., Duncan C.J.G., Pasco D.S. (2000). A new indanone from the marine cyanobacterium *Lyngbya majuscula* that inhibits hypoxia-induced activation of the VEGF promoter in Hep3B cells. J. Nat. Prod..

[8] Yoshihira K., Fukuoka M., Kuroyanagi M., Natori S. (1971). 1-Indanone Derivatives from Bracken, *Pteridium aquilinum* var. latiusculum. Chem. Pharm. Bull..

[9] Saraux N., Cretton S., Kilicaslan O.S., Occioni C., Ferro A., Quirós-Guerrero L., Karimou S., Christen P., Cuendet M. (2022). Isolation and Structure Elucidation of Compounds from *Sesamum alatum* and Their Antiproliferative Activity against Multiple Myeloma Cells. J. Nat. Prod..

[10] Ito T., Tanaka T., Iinuma M., Nakaya K., Takahashi Y., Sawa R., Murata J., Darnaedi D. (2004). Three new resveratrol oligomers from the stem bark of *Vatica pauciflora*. J. Nat. Prod..

[11] Ge X., Ye G., Li P., Tang W.J., Gao J.L., Zhao W.M. (2008). Cytotoxic diterpenoids and sesquiterpenoids from *Pteris multifida*. J. Nat. Prod..

[12] Teruya T., Nakagawa S., Koyama T., Suenaga K., Kita M., Uemura D. (2003). Nakiterpiosin, a novel cytotoxic C-nor-D-homosteroid from the Okinawan sponge *Terpios hoshinota*. Tetrahedron Lett..

[13] Nakashima T., Boonsnongcheep P., Kimura T., Iwatsuki M., Sato N., Nonaka K., Prathanturarug S., Takahashi Y., Omura S. (2015). New compounds, nanaomycin F and G, discovered by physicochemical screening from a culture broth of *Streptomyces rosa* subsp. notoensis OS-3966. J. Biosci. Bioeng..

[14] Zhao Q., Chen G.D., Feng X.L., Yu Y., He R.R., Li X.X., Huang Y., Zhou W.X., Guo L.D., Zheng Y.Z. (2015). Nodulisporiviridins A-H, Bioactive Viridins from *Nodulisporium* sp.. J. Nat. Prod..

[15] Yang L., Qin L.H., Bligh S.W.A., Bashall A., Zhang C.F., Zhang M.A., Wang Z.T., Xu L.S. (2006). A new phenanthrene with a spirolactone from *Dendrobium chrysanthum* and its anti-inflammatory activities. Bioorg. Med. Chem..

[16] Wang B., Zhao Y.J., Zhao Y.L., Liu Y.P., Li X.N., Zhang H.B., Luo X.D. (2020). Exploring Aporphine as Anti-inflammatory and Analgesic Lead from *Dactylicapnos scandens*. Org. Lett..

[17] Chen D.L., Chen M.Y., Hou Y., Wang C.H., Sun Z.C., Yang Y., Liang H.Q., Ma G.X., Xu X.D., Wei J.H. (2022). Cadinane-Type Sesquiterpenoids with Cytotoxic Activity from the Infected Stems of the Semi-mangrove *Hibiscus tiliaceus*. J. Nat. Prod..

[18] Kawamoto Y., Noguchi N., Kobayashi T., Ito H. (2023). Total Synthesis of Lucidumone through Convenient One-pot Preparation of the Tetracyclic Skeleton by Claisen Rearrangement and Subsequent Intramolecular Aldol Reaction. Angew. Chem. Int. Edit..

[19] Lan X., Guo S., Zhao Y., Zhang M., Zhang D., Leng A., Ying X. (2023). A novel skeleton alkaloid from *Portulaca oleracea* L. and its bioactivities. Fitoterapia.

[20] Hayakawa Y., Kobayashi T., Izawa M. (2013). Indanostatin, a new neuroprotective compound from *Streptomyces* sp.. J. Antibiot..

[21] Liang W.Q., Xu G.J., Weng D., Gao B., Zheng X.F., Qian Y. (2015). Anti-Osteoporotic Components of *Rubus chingii*. Chem. Nat. Compd..

[22] Amatov T., Jangra H., Pohl R., Cisarova I., Zipse H., Jahn U. (2018). Unique Stereoselective Homolytic C-O Bond Activation in Diketopiperazine-Derived Alkoxyamines by Adjacent Amide Pyramidalization. Chem. Eur. J..

[23] Wattana-Amorn P., Charoenwongsa W., Williams C., Crump M.P., Apichaisataienchote B. (2016). Antibacterial activity of cyclo(L-Pro-L-Tyr) and cyclo(D-Pro-L-Tyr) from *Streptomyces* sp. strain 22-4 against phytopathogenic bacteria. Nat. Prod. Res..

[24] Xiang W.X., Liu Q., Li X.M., Lu C.H., Shen Y.M. (2020). Four pairs of proline-containing cyclic dipeptides from *Nocardiopsis* sp. HT88, an endophytic bacterium of *Mallotus nudiflorus* L.. Nat. Prod. Res..

[25] Phongsopitanun W., Kanchanasin P., Khanboon A., Pittayakhajonwut P., Suwanborirux K., Tanasupawat S. (2021). Marine *Streptomyces chumphonensis* KK1-2^T^ produces piericidin A1 as the major secondary metabolite. Sci. Asia.

[26] Bruhn T., Schaumlöffel A., Hemberger Y., Pescitelli G. (2017). SpecDis Version 1.71, Berlin, Germany. http://specdis-software.jimdo.com.

[27] Nagahawatta D.P., Liyanage N.M., Jayawardhana H., Lee H.G., Jayawardena T.U., Jeon Y.J. (2022). Anti-Fine Dust Effect of Fucoidan Extracted from *Ecklonia maxima* Leaves in Macrophages via Inhibiting Inflammatory Signaling Pathways. Mar. Drugs.

[28] Nagahawatta D.P., Lee H.G., Liyanage N.M., Jayawardhana H.H.A.C.K., Wang L., Kim H.S., Jeon Y.J. (2023). Alginic acid, a functional dietary ingredient derived from *Ecklonia maxima* stipe, attenuates the pro-inflammatory responses on particulate matter-induced lung macrophages. J. Funct. Foods.

[29] Ko E.Y., Cho S.H., Kwon S.H., Eom C.Y., Jeong M.S., Lee W., Kim S.Y., Heo S.J., Ahn G., Lee K.P. (2017). The roles of NF-*κ*B and ROS in regulation of pro-inflammatory mediators of inflammation induction in LPS-stimulated zebrafish embryos. Fish Shellfish Immunol..

