# Supplementary material for: Streptinone, a New Indanone Derivative from a Marine-Derived Streptomyces massiliensis, Inhibits Particulate Matter-Induced Inflammation
Source: Mar Drugs. 2023 Dec 14;21(12):640. doi: 10.3390/md21120640 (PMC10744446; doi:10.3390/md21120640)
Supplement: Supplementary file 1 [file marinedrugs-21-00640-s001.zip › marinedrugs-2774016-supplementary.pdf]

Supporting Information

Streptinone, a New Indanone Derivative from a Marine-Derived *Streptomyces massiliensis*, Inhibits Particulate Matter-Induced Inflammation

Hwa-Sun Lee [1,2], Dineth Pramuditha Nagahawatta [3], You-Jin Jeon [3], Min Ah Lee [1,2], Chang-Su Heo [1,4], Sun Joo Park [2], and Hee Jae Shin [1,4,*]

Contents

Figure S1. ^{1}H NMR spectrum of compound **1** (600 MHz, CD_3OD). S2

Figure S2. ^{13}C NMR spectrum of compound **1** (150 MHz, CD_3OD). S2

Figure S3. HSQC spectrum of compound **1** in CD_3OD. S3

Figure S4. COSY spectrum of compound **1** in CD_3OD. S3

Figure S5. HMBC spectrum of compound **1** in CD_3OD. S4

Figure S6. 1D selective NOESY spectrum of compound **1** in CD_3OD (irradiated at H-9) S4

Figure S7. 1D selective NOESY spectrum of compound **1** in CD_3OD (irradiated at H-8-Me). S5

Figure S8. HR-ESIMS spectrum of compound **1**. S6

Figure S9. ^{1}H NMR spectrum of cyclo (Gly-L-Leu) (**2**) (600 MHz, CD_3OD). S7

Figure S10. ^{1}H NMR spectrum of cyclo (L-Tyr-D-Pro) (**3**) (600 MHz, CD_3OD). S7

Figure S11. ^{1}H NMR spectrum of cyclo (L-Phe-D-Pro) (**4**) (600 MHz, CD_3OD). S8

Figure S12. ^{1}H NMR spectrum of piericidin A (**5**) (600 MHz, $CDCl_3$). S8

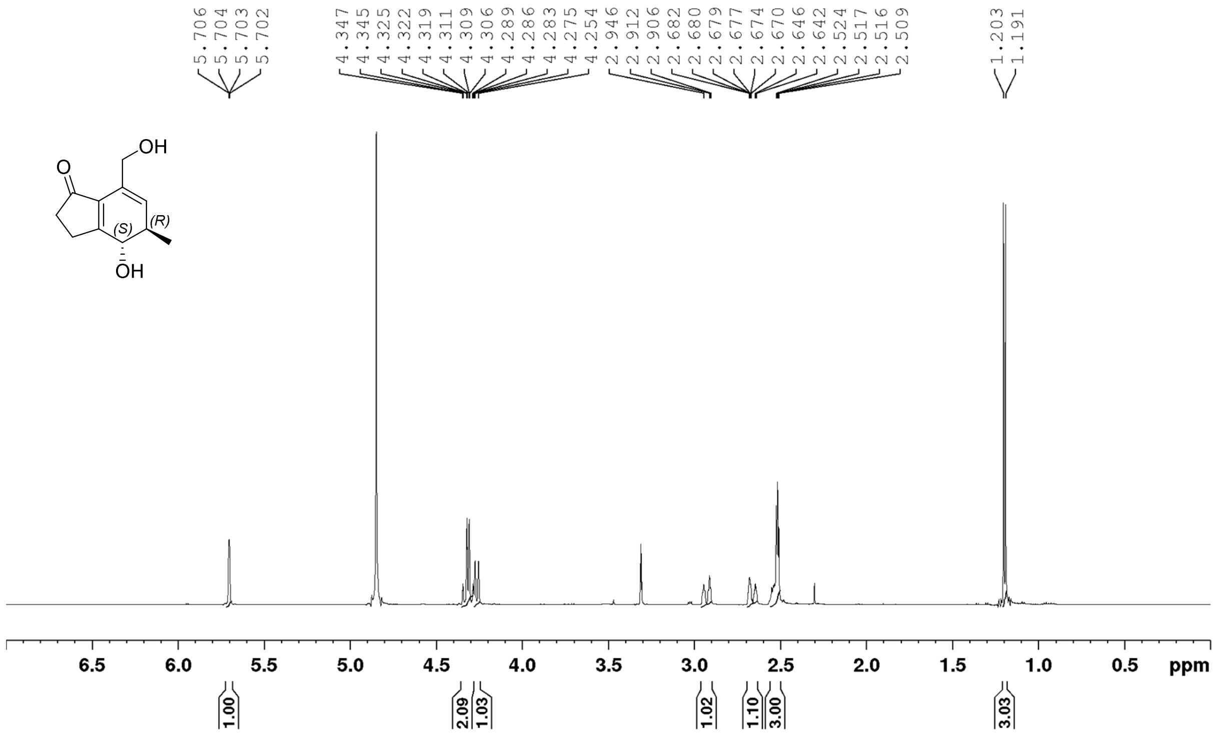

Figure S1. ^{1}H NMR spectrum of compound **1** (600 MHz, CD_3OD).

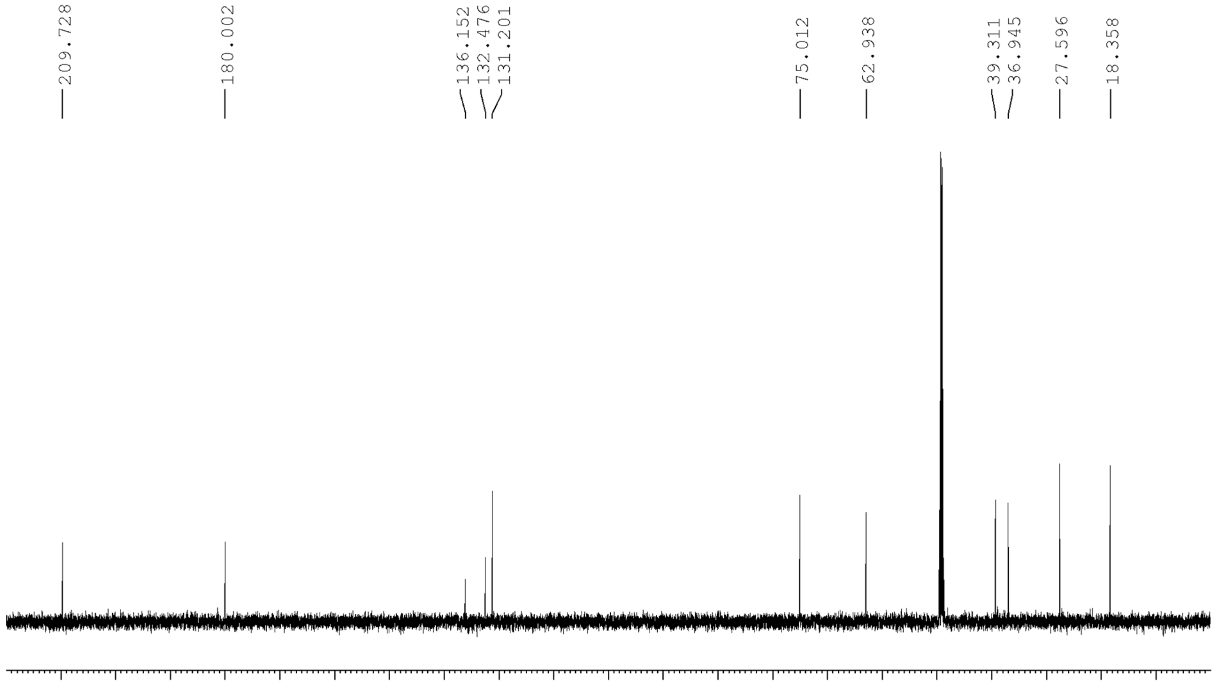

Figure S2. ^{13}C NMR spectrum of compound **1** (150 MHz, CD_3OD).

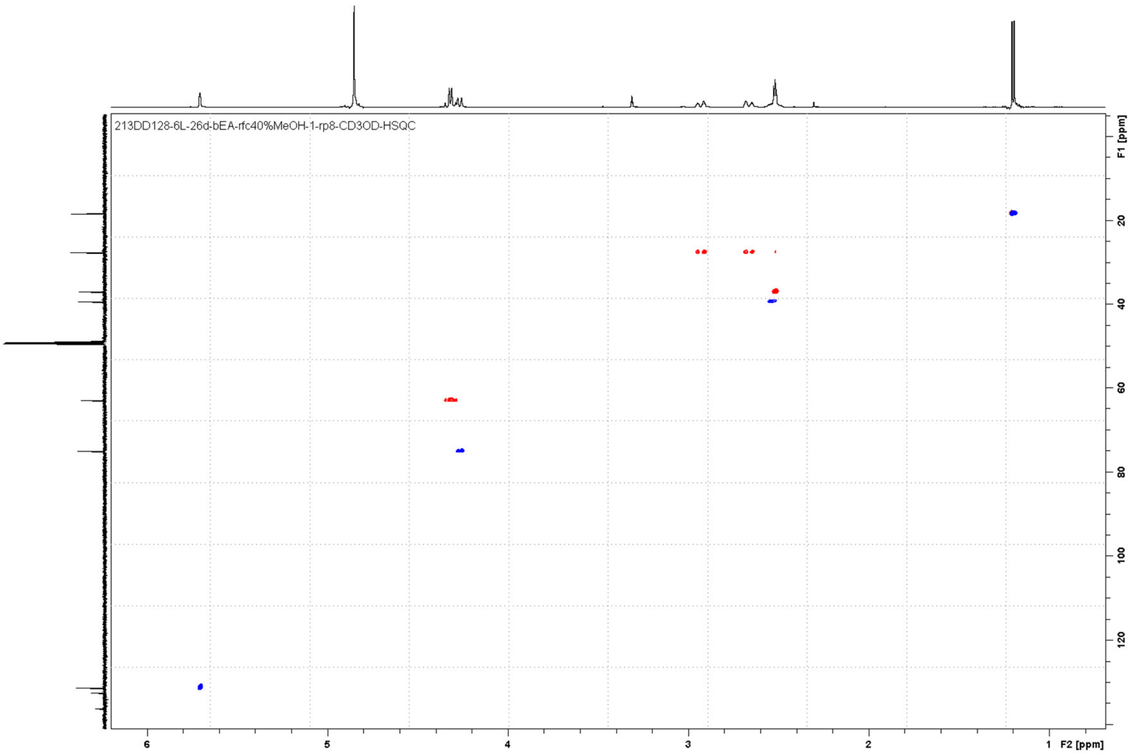

Figure S3. HSQC spectrum of compound **1** in CD_3OD.

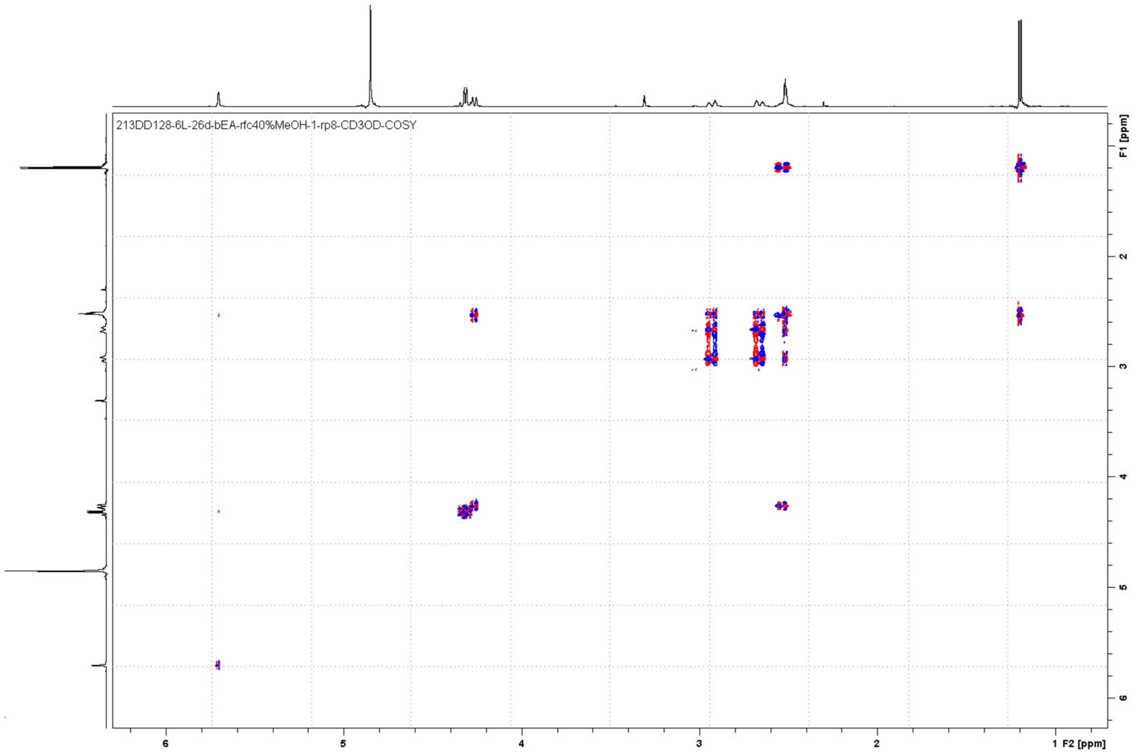

Figure S4. COSY spectrum of compound **1** in CD_3OD.

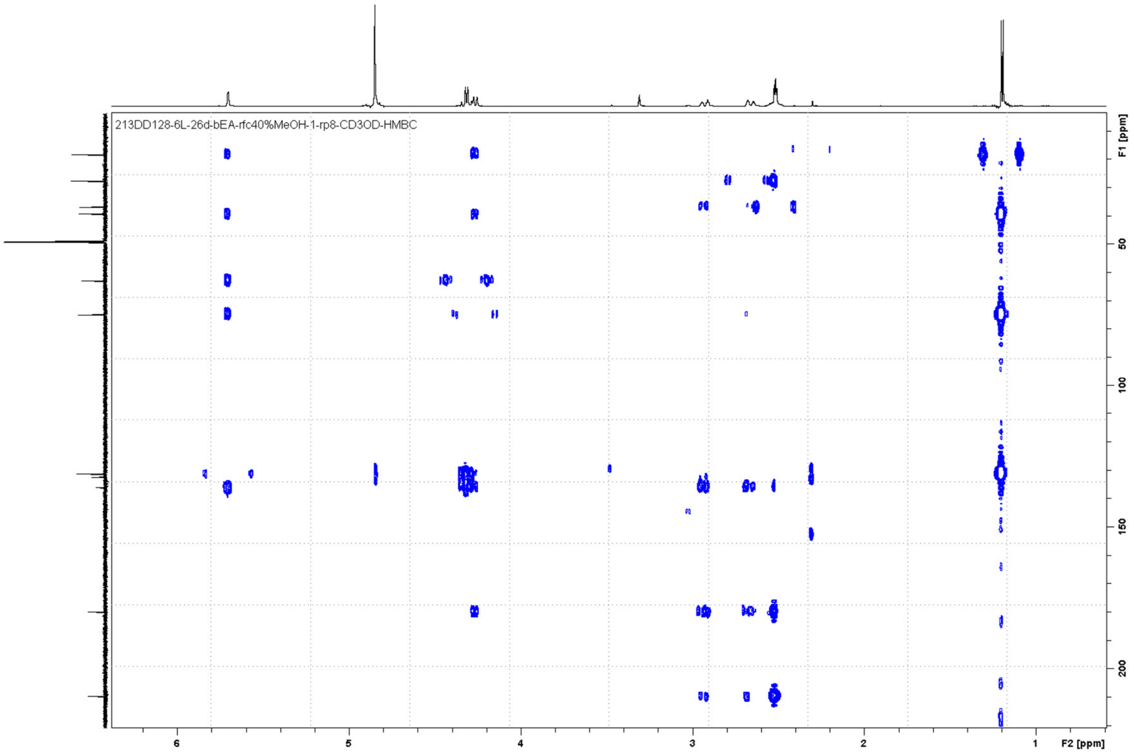

Figure S5. HMBC spectrum of compound **1** in CD_3OD.

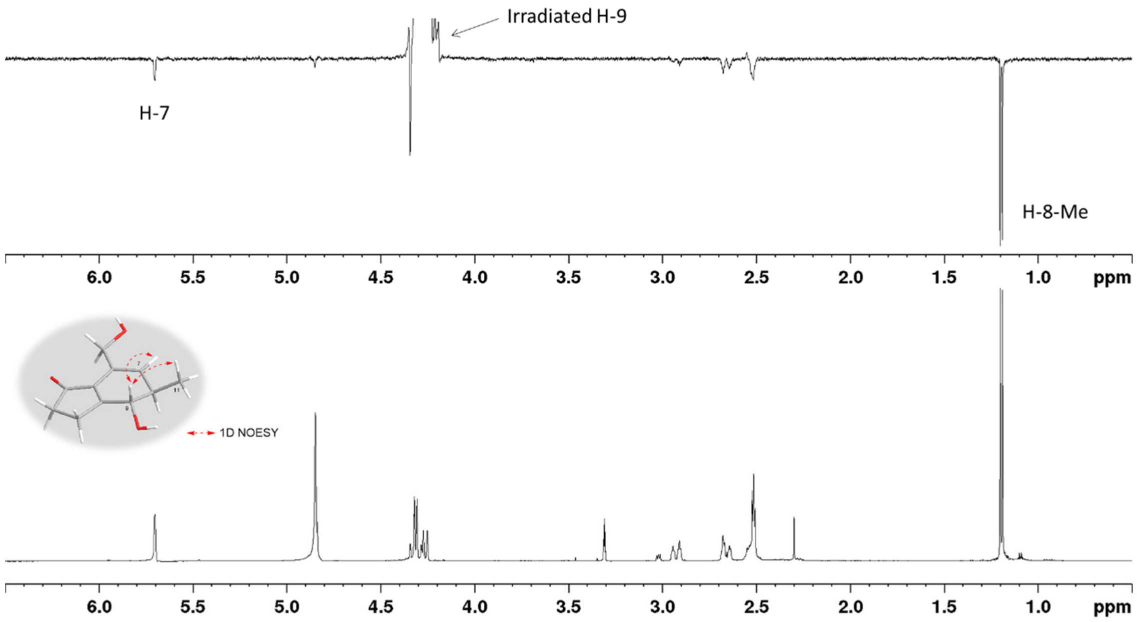

Figure S6. 1D selective NOESY spectrum of compound **1** in CD_3OD (irradiated at H-9).

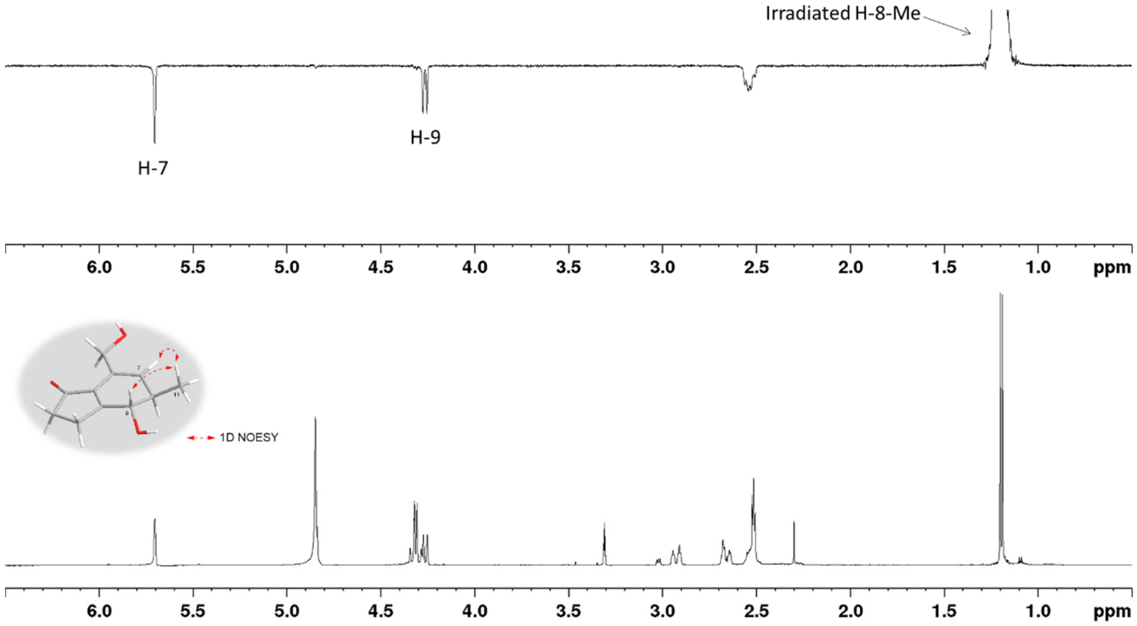

Figure S7. 1D selective NOESY spectrum of compound **1** in CD_3OD (irradiated at H-8-Me).

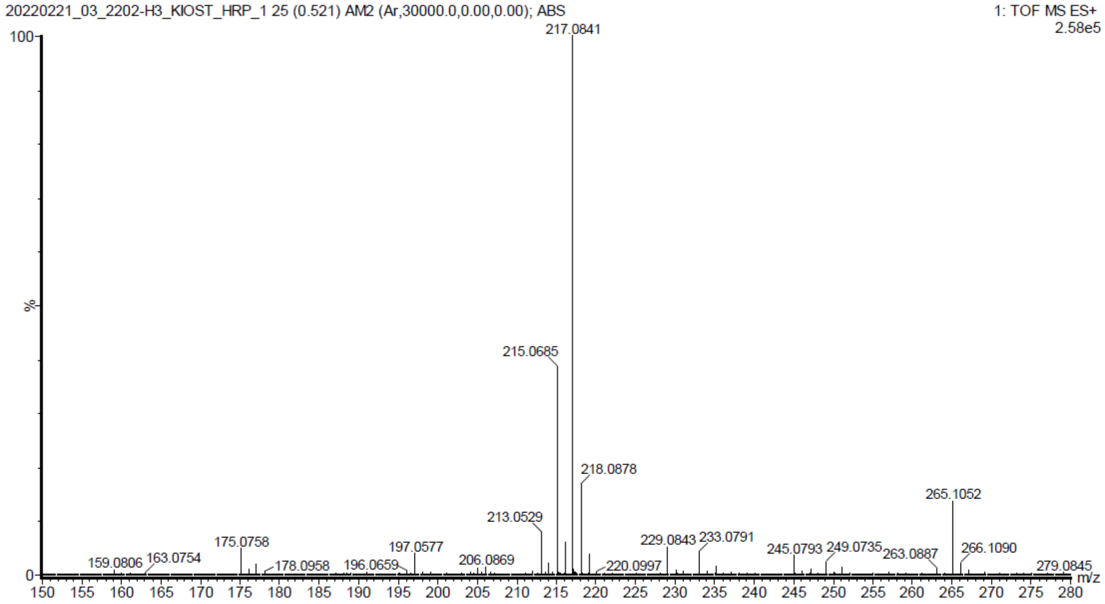

Elemental Composition Report

Single Mass Analysis
Tolerance = 5.0 PPM / DBE: min = -3.0, max = 200.0
Element prediction: Off
Number of isotope peaks used for i-FIT = 3

Monoisotopic Mass, Even Electron Ions
Elements Used:
C: 0-31 H: 0-50 O: 0-7 Na: 0-1

Minimum:				-3.0				
Maximum:		300.0	5.0	200.0				
Mass	Calc. Mass	mDa	PPM	DBE	i-FIT	Norm	Conf(%)	Formula
217.0841	217.0841	0.0	0.0	4.5	1193.0	n/a	n/a	C11 H14 O3 Na
215.0685	215.0684	0.1	0.5	5.5	1197.9	n/a	n/a	C11 H12 O3 Na

Figure S8. HR-ESIMS spectrum of compound **1**.

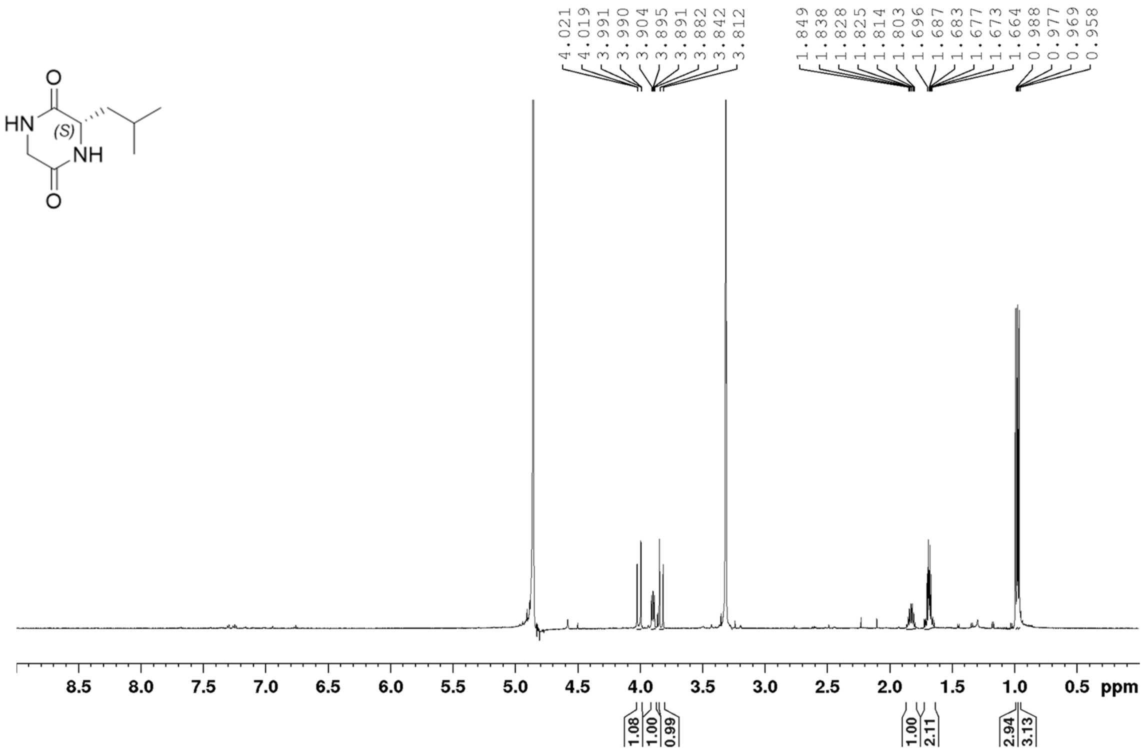

Figure S9. ^{1}H NMR spectrum of cyclo (Gly-L-Leu) (**2**) (600 MHz, CD_3OD).

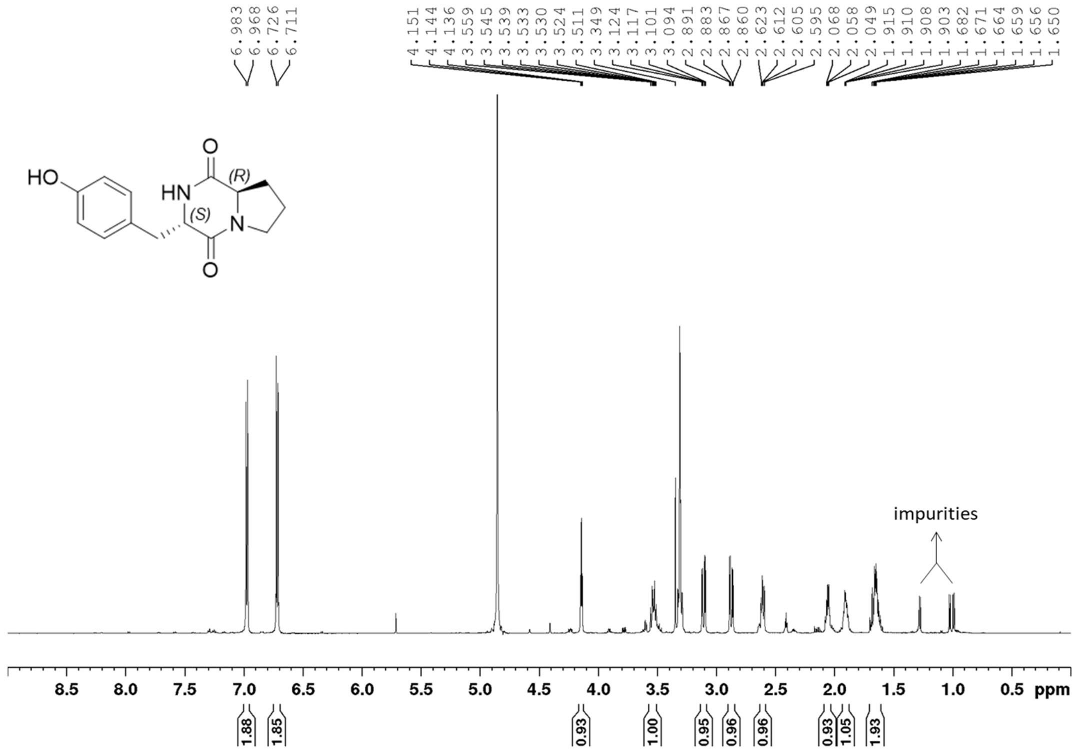

Figure S10. ^{1}H NMR spectrum of cyclo (L-Tyr-D-Pro) (**3**) (600 MHz, CD_3OD).

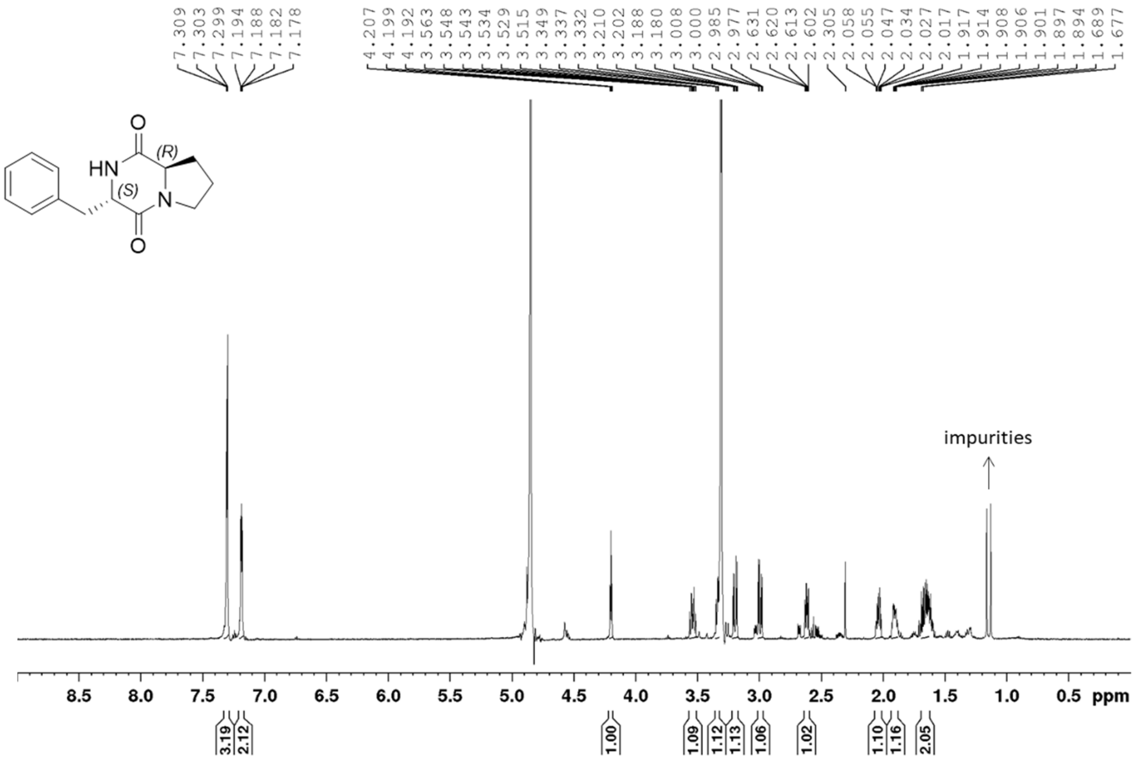

Figure S11. ^{1}H NMR spectrum of cyclo (L-Phe-D-Pro) (**4**) (600 MHz, CD_3OD).

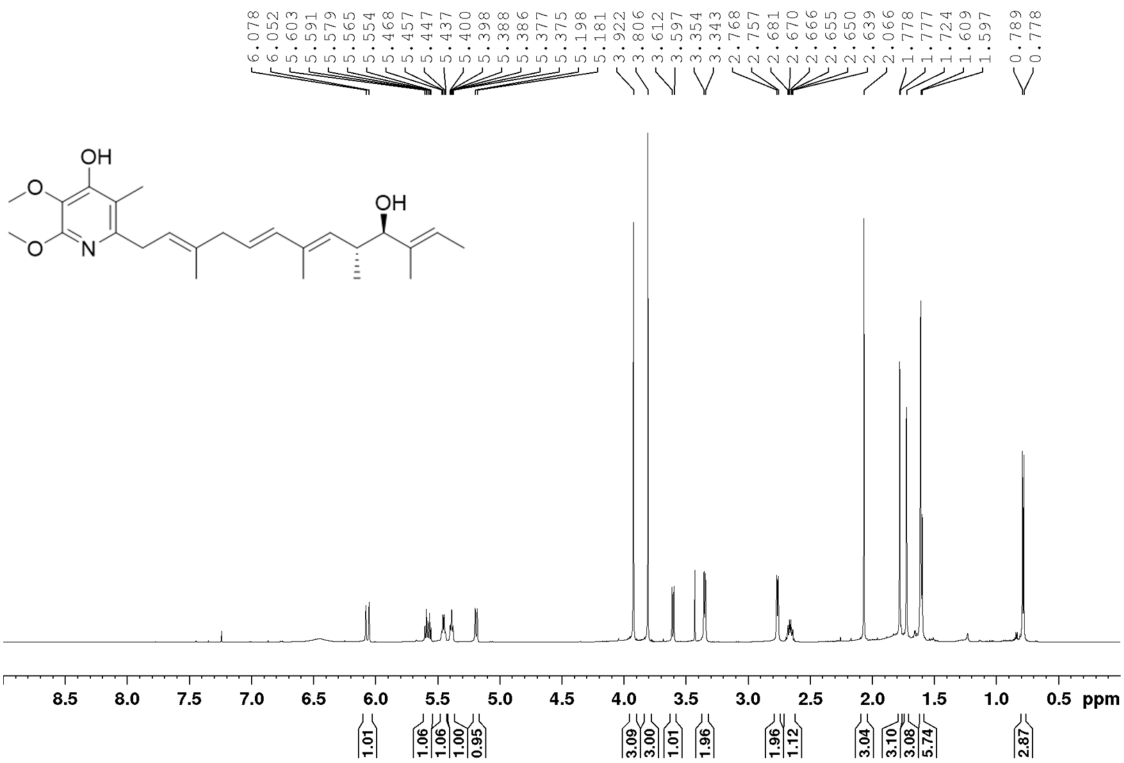

Figure S12. ^{1}H NMR spectrum of piericidin A (**5**) (600 MHz, $CDCl_3$).
